# Prevalence and Associated Risk Factors of Chronic Kidney Disease in an Elderly Population from Eastern China

**DOI:** 10.3390/ijerph16224383

**Published:** 2019-11-09

**Authors:** Andong Ji, Chunlei Pan, Hongxia Wang, Zhezhen Jin, Joseph H. Lee, Qincheng Wu, Qixiao Jiang, Lianhua Cui

**Affiliations:** 1 School of Public Health, Medical College of Qingdao University, Qingdao 266021, Shandong Province, China; 2017021617@qdu.edu.cn (A.J.); jiangq@qdu.edu.cn (Q.J.); 2016020269@qdu.edu.cn (Q.W.); 2 Health Center of Liuting Street, Chengyang District, Qingdao 266108, Shandong Province, China; lt776@126.com (C.P.); weisheng4513@126.com (H.W.); 3 Department of Biostatistics, Mailman School of Public Health, Columbia University, New York, NY 10032, USA; zj7@cumc.columbia.edu; 4 Sergievsky Center, Taub Institute, and Department of Epidemiology, Mailman School of Public Health, Department of Neurology, College of Physicians and Surgeons, Columbia University, New York, NY 10032, USA; JHL2@cumc.columbia.edu

**Keywords:** chronic kidney disease, elderly, prevalence, epidemiology

## Abstract

Chronic kidney disease (CKD) is a global major public health problem. Almost all of previous studies evaluating the prevalence of CKD focused on adults, while studies among the elderly were relatively rare, especially in China. The aim of this study was to investigate the prevalence and associated risk factors of CKD among the elderly in Qingdao, China. This was a cross-sectional study with 38,038 inhabitants (aged 60–109) randomly recruited in Qingdao, China. All participants were required to complete a questionnaire for their demographic characteristics. Blood and urine samples of participants were collected, and the albumin and creatinine levels were measured for albuminuria and estimated glomerular filtration rate (eGFR) assessment. The associations between risk factors and indicators of kidney damage were analyzed by logistic regression. A total of 34,588 inhabitants completed the survey. The overall prevalence of CKD was 11.41% (95% confidence interval (CI): 11.07–11.74%) in the elders from Qingdao in 2016. The prevalence of albuminuria and low eGFR (<60 mL/min per 1·73 m²) were 8.47% (95% CI: 8.17–8.76%) and 3.98% (95% CI: 3.78–4.19%), respectively. Older age, hypertension, diabetes, anemia, hyperuricemia, hyperhomocysteinemia, hypertriglyceridemia, obesity, and LDL-C ≥ 4.1 mmol/L were independently associated with the presence of CKD. In conclusion, common chronic non-communicable diseases, including hypertension, diabetes, obesity, hyperhomocysteinemia, hyperuricemia, and hypertriglyceridemia, were associated with greater prevalence of CKD.

## 1. Introduction

Chronic kidney disease (CKD) has received increasing attention as a major public health problem around the world [1]. The burden of CKD was not only reflected in the needs for life-long dialysis or renal replacement therapy when entering end-stage renal disease (ESRD), but also highlighted in association with a higher risk of morbidity (especially due to cardiovascular disease), mortality, hospitalization, and cognitive dysfunction. In 2010, the estimated number of patients receiving dialysis was 327,000 (442 pmp: per million people) in Europe, and the number was 441,000 (1273 pmp) and 909,000 (218 pmp) in North America and Asia, respectively [2]. In China, the prevalence of dialysis was 33.2 pmp in 1999, and the number had surged to 402.18 pmp in 2015, and the corresponding number of hemodialysis patients was approximately 553,000 [3]. A similar trend was also present in other developing countries. Furthermore, patients with CKD generally had relatively higher medical expenditure comparing with those who had other comorbidities [3].

Recently, with the rapid development of China’s economy, the issue of population aging is becoming more prominent. The 2010 census data of China indicated that the population aged 60 or older accounted for 13.26% of the total population, which was 2.93% higher than that in 2000 [4]. The burden of CKD is likely to escalate with aging [5,6]. The 2015 Annual Data Report of the China Kidney Disease Network showed that nearly half of the patients with CKD were aged 60 years or older [3]. Levey et al. also pointed out that CKD affected more than half of people older than 70 years [7]. Both findings suggested that the elderly population is a high-risk group for CKD. Moreover, early stages of CKD generally have few symptoms and older people are at greater risk of complications and death. Therefore, it is necessary to pay close attention to the elderly population to reduce their CKD burden. So far, many studies [5,8,9,10] have been conducted around the world to investigate the prevalence of CKD. However, almost all of those studies were focused on adults, while studies within the elderly population were relatively rare, especially in China.

Qingdao is an important coastal city in eastern China with a population of 9.29 million. To the best of our knowledge, this is the first study to explore the prevalence of CKD and its risk factors among the elderly in China according to KDIGO (Kidney Disease: Improving Global Outcomes)’s CKD guideline [11]. The results of the present study will help to identify appropriate interventional strategies for burden reduction in elderly patients with CKD.

## 2. Materials and Methods

### 2.1. Study Population

This was a cross-sectional study that aimed to determine the burden of CKD and its related risk factors in the elderly population in Qingdao from January to December 2016. A required number of participants were selected using a multistage, stratified sampling method. In the first stage, the Laoshan and Chengyang district were randomly selected in Qingdao. In the second stage, we selected five streets (Jinjialing, Zhonghan, Shazikou, Wanggezhuang, and Beizhai from Laoshan; Chengyang, Xifu, Liuting, Xiazhuang, and Shangma from Chengyang) from each district. In the third stage, 15 to 20 communities were randomly selected from each of street. In the final stage, individuals were randomly chosen from each community. All participants were required to fulfill the following conditions: (1) aged ≥60 years; (2) resided in the area for more than 5 years; and (3) without malignant tumor or mental disorder.

Eventually, a total of 38,038 subjects aged 60 years or older were selected and invited to participate in the present study. Among them, 34,588 subjects completed the survey and examination. The response rate was 90.9%. The study protocol was approved by the ethics committees of Qingdao University (Qingdao, China, ethical approved project identification code: 20151103). All participants provided their written informed consent before they participated in the study.

### 2.2. Screening Protocol and Evaluation Criteria

All study investigators and staff members completed a training program about the operation protocol of the study. Data collection was conducted in examination centers at local health stations or community clinics in the participants’ residential area. All participants completed a questionnaire documenting their demographic status (e.g., age and sex), personal and family health history (e.g., hypertension and diabetes), and lifestyle behavior (e.g., exercise) with the assistance of trained professionals. Anthropometric measurements, such as weight, height, and waist circumference, were measured by standard equipment for each subject after removal of shoes and heavy clothing. The body mass index (BMI) was calculated as weight (in kilograms) divided by height squared (in square meters). Venous blood samples were collected after an overnight fast of at least 10 h for determination of various biomarkers. A morning spot urine specimen was collected for albumin and creatinine analysis.

### 2.3. Albuminuria

Urinary albumin and creatinine were detected from a fresh morning urine sample stored at 4 °C for less than 1 week. Urinary albumin was measured with immunoturbidimetric tests. Urinary creatinine was measured via Jaffe’s kinetic method on a Hitachi 7600 autoanalyzer (Hitachi, Tokyo, Japan). The urinary albumin to creatinine ratio (ACR; mg/g creatinine) was calculated, and patients with an ACR greater than 30 mg/g were regarded as having albuminuria [7].

### 2.4. Estimated Glomerular Filtration Rate (eGFR)

The measurement of Serum creatinine (Scr) was carried out using the identical method as urinary creatinine on a Hitachi 7600 autoanalyzer (Hitachi, Tokyo, Japan). eGFR was calculated using the simplified modification of diet in renal disease (MDRD) equation on the basis of data from Chinese CKD patients [12]. Reduced renal function was defined as eGFR < 60 mL/min per 1·73 m²:

eGFR (mL/min per 1·73 m²) = 175 × Scr^−^¹·²³⁴ × age^−^⁰·¹⁷⁹ × 0·79 (if female),

where Scr is serum creatinine concentration (in mg/dL) and age in years.

### 2.5. Hypertension Status

Blood pressure was measured with a mercury sphygmomanometer, three times at 5 min intervals, after participants had sat and rested for at least 15 min. The mean values of the three measurements were calculated, unless the difference between the readings was greater than 10 mm Hg, in which case the mean of the two closest of the three readings was used. Hypertension was defined as a systolic blood pressure (SBP) ≥ 140 mm Hg or diastolic blood pressure (DBP) ≥ 90 mm Hg, or any administration of antihypertensive medications in the past 2 weeks regardless of blood pressure or any self-reported history of hypertension [13].

### 2.6. Diabetes Status

Fasting blood glucose was determined with a glucose oxidase enzymatic method. Participants with fasting plasma glucose ≥ 7·0 mmol/L or any administration of oral hypoglycemic agents or any self-reported history of diabetes was categorized as diabetes [14].

### 2.7. Other Measurements

Hemoglobin, uric acid, homocysteine, serum total cholesterol, triglyceride, low-density lipoprotein cholesterol (LDL-C), and high-density lipoprotein cholesterol (HDL-C) were measured with a Hitachi 7600 automatic analyzer (Hitachi, Tokyo, Japan). Plasma homocysteine concentration >15 μmol/L was recognized as hyperhomocysteinemia (HHcy) [15]. Serum uric acid levels >422 μmol/L for men and >363 μmol/L for women were considered hyperuricemia (HUA) [16]. Anemia was defined as hemoglobin <130 g/L for men and women older than 50 years and <120 g/L for women 50 years or younger [17]. Subjects were considered to have central obesity if the waist circumference was 90 cm or greater for men and 80 cm or greater for women [18]. BMI of 28 or greater was categorized as obesity [18]. Subjects with serum total cholesterol >6.2 mmol/L or serum triglyceride >2.3 mmol/L at screening were noted to have hypercholesteremia [19] or hypertriglyceridemia [20], respectively.

### 2.8. Statistical Analysis

Data analysis was performed with SPSS 17.0 software (SPSS Inc, Chicago, IL, USA) and all statistical tests were two-sided at the significance level 0.05. Continuous variables were expressed as mean ± SD and categorical variables presented as proportion. Baseline characteristics were described and compared between participants with or without indicators of CKD. The prevalence of CKD indicators (albuminuria and eGFR < 60 mL/min per 1.73 m^2^) and CKD was reported and compared among sexes, age, and comorbidities groups. The overall CKD prevalence, defined as eGFR less than 60 mL/min per 1.73 m^2^ or markers of kidney damage (e.g., albuminuria) [7], was estimated. Differences between subjects were analyzed with two-tailed unpaired student’s t tests for continuous data and by chi-square test for categorical data. Multivariate logistic regression analyses were performed to access the association between various potential risk factors and the prevalence of albuminuria, reduced kidney function, and CKD, with adjustment for any confounding variables.

## 3. Results

### 3.1. Demographic Characteristics of the Participants

38,038 participants were recruited into this study and 34,588 completed the survey and examination, in which 43.3% were male and 56.7% were female. Their ages ranged from 60 to 109 years, with the mean age of 71.0 ± 6.7 years. Among all the 34,588 participants, the prevalence of obesity, central obesity, diabetes, hypertension, anemia, hyperuricemia, and hyperhomocysteinemia were 25.7%, 56.3%, 24.8%, 70.6%, 18.6%, 23.4%, and 15.4%, respectively. Participants with low eGFR or albuminuria were older, more likely to be female, and had higher prevalence of obesity, central obesity, hypertension, diabetes, anemia, hyperuricemia, and hyperhomocysteinemia, than those without indicators of CKD (Table 1). Among all hypertensive subjects, 62.5% noted that they had hypertension but only 43.4% of all the hypertensives took antihypertensive medications regularly. Diabetes awareness was 66.1% among all studied subjects but only 45.8% of them had treatment.

### 3.2. Albuminuria

**Figure 1:**
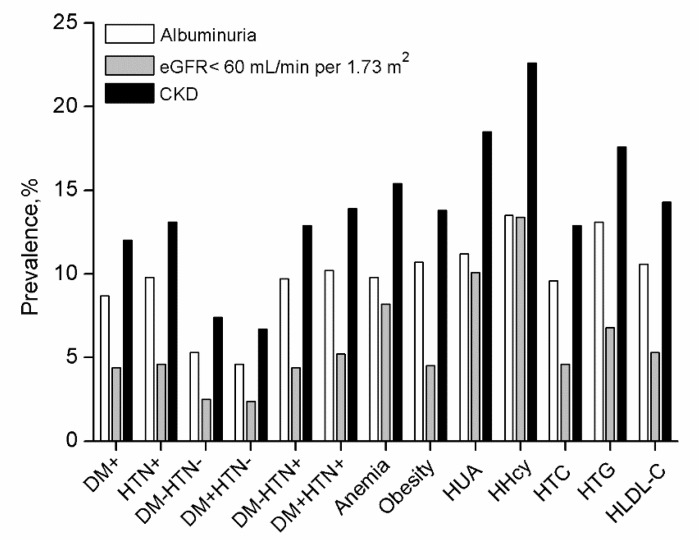
Prevalence of indicators of chronic kidney disease in different comorbidities groups. DM, diabetes; HTN, hypertension; HUA, hyperuricemia; HHcy, hyperhomocysteinemia; HTC, hypercholesteremia; HTG, hypertriglyceridemia; HLDL-C, LDL-C ≥ 4.1 mmol/L. CKD, chronic kidney disease.

Among all the participants, 8.47% (95% CI, 8.17%–8.76%) had albuminuria (Table 2). No gender differences were observed in the prevalence of albuminuria (8.51% versus 8.41%; *p* = 0.747), and an increasing trend in the prevalence of albuminuria with advanced ages was seen in both genders (*p* < 0.001 for females, *p* < 0.001 for males across age groups). Of all the participants with albuminuria, 25.4% (n = 745) had diabetes, 82.1% (n = 2406) had hypertension, 30.8% (n = 902) had hyperuricemia, and 24.6% (n = 721) had hyperhomocysteinemia (Table 1). The highest prevalence of albuminuria was observed in subjects with hyperhomocysteinemia (13.52%); whereas in subjects with diabetes without hypertension, the prevalence of albuminuria was only 4.6% (Figure 1).

### 3.3. Estimated GFR < 60 mL/min per 1.73 m^2^

The prevalence of reduced kidney function (estimated GFR < 60 mL/min per 1.73 m^2^) was 3.98% (95% CI, 3.78%–4.19%). The prevalence was greater in females than in males (4.52% versus 3.27%; *p* < 0.001), and an increasing trend in the prevalence of reduced kidney function with advanced ages was observed in both genders (*p* < 0.001 for females, *p* < 0.001 for males across age groups). Of all participants with eGFR < 60 mL/min per 1.73 m^2^, 27.5% (n = 378) had diabetes, 81.8% (n = 1126) had hypertension, 59.3% (n = 817) had hyperuricemia, and 51.7% (n = 712) had hyperhomocysteinemia (Table 1). The highest prevalence of eGFR < 60 mL/min per 1.73 m^2^ was observed in subjects with hyperhomocysteinemia (13.36%); whereas in subjects with diabetes without hypertension, prevalence of eGFR < 60 mL/min per 1.73 m^2^ was only 2.4%.

### 3.4. Prevalence of CKD

**Figure 2:**
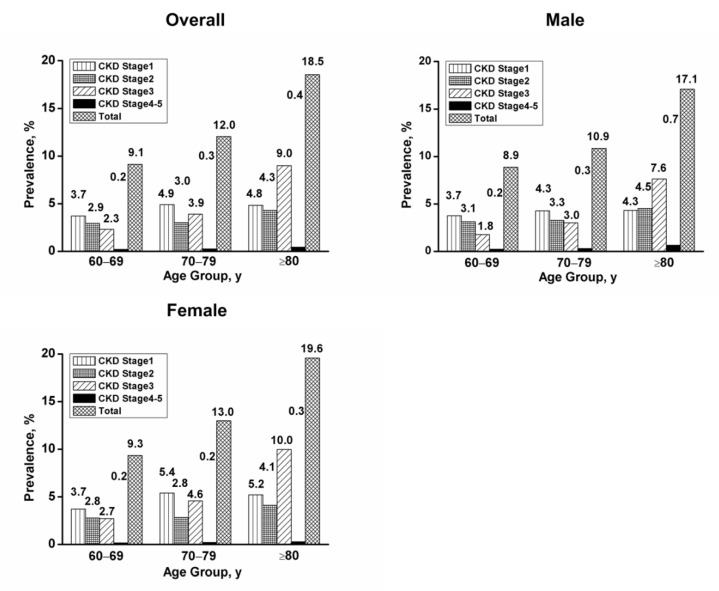
Prevalence of chronic kidney disease in different age groups. CKD—chronic kidney disease.

A total of 11.41% (95% CI, 11.07%–11.74%) of studied participants (n = 34,588) had CKD and those were predominantly in CKD stages 1 to 3 (4.29% were at stage 1, 3.13% were at stage 2, and 3.74% were at stage 3) (Table 2). The prevalence of CKD was significantly higher in females than in males (12% versus 10.63%, *p* < 0.001), which increased in both genders with age (*p* < 0.001 for females and males across age groups). The CKD prevalence was 9.13% for the group of 60–69 years old, 12.04% for 70–79 years old, and 18.53% for 80 years and older (Table 3). The highest prevalence of CKD was observed in subjects with hyperhomocysteinemia (22.64%); whereas in subjects with diabetes without hypertension, prevalence of CKD was only 6.7% (Table 3 and Figure 1). Figure 2 showed the prevalence of CKD in different stages among different age groups. It is obvious that the prevalence of CKD was greater in females than males. Additionally, the stage distribution of CKD prevalence changed significantly, which the prevalence of stages 3 to 5 CKD increased with age.

### 3.5. Multivariate Analyses

The results of the multivariable logistic regression model showed the differential association of risk factors with decreased eGFR, albuminuria, and CKD (Table 4). For decreased eGFR, albuminuria, and CKD, common risk factors of older age (increased by 10 years), hypertension, diabetes, anemia, hyperuricemia, hyperhomocysteinemia, and hypertriglyceridemia were shared. Obesity and LDL-C ≥ 4.1 mmol/L were independently associated with albuminuria and CKD. Being female was a significant risk factor for decreased eGFR, whereas being male was associated with albuminuria. Additionally, exercise was a protective factor for decreased eGFR, albuminuria, and CKD. Notably, adjusted ORs of hyperuricemia and hyperhomocysteinemia for decreased eGFR was abnormally high (3.992, 95% CI = 3.541–4.499; 5.645, 95% CI = 4.971–6.409, respectively).

## 4. Discussion

To the best of our knowledge, this is the first community-based study with large sample size to explore CKD prevalence and its risk factors among the elderly population in China. Our results indicated that at least one indicator of kidney damage was observed among 11.41% of elderly people 60 years or more in Qingdao. Notable gender variation in the kidney damage was observed, with a higher prevalence significantly tilted toward the female gender. Moreover, the CKD prevalence increased in both genders with advanced ages. Older age, hypertension, diabetes, anemia, hyperuricemia, hyperhomocysteinemia, hypertriglyceridemia, obesity, and LDL-C ≥ 4.1 mmol/L accounted for higher prevalence of CKD.

Previous studies in China evaluating the prevalence of CKD mainly focused on adults (18 years or older). For example, three studies reported the estimations of CKD prevalence among adults 18 years or older were 11.8% (Shanghai) [17], 13% (Beijing) [21], and 19.1% (Tibet) [18], respectively. In Guangzhou, 12.1% residents 20 years or older had at least one indicator of kidney damage [22]. In Beijing, 11.3% adults older than 40 years had CKD [23]. The situation was similar in other developing countries [24,25,26]. Compared with previous studies, our study provides unique reference data for better understanding of the CKD burden in elderly population. Unlike previous studies in China, the present study did not include hematuria as an indicator of kidney damage, which may underestimate the prevalence of CKD in this study population.

A new MDRD equation, which was modified based on data from Chinese CKD patients, was introduced for GFR assessments. In the present study, the prevalence of reduced renal function was estimated to be 3.98%, which was similar to the results from Guangzhou subjects aged 20 or older (3.2%) [22]. However, the prevalence varied greatly among different age groups in other cities of China, such as Beijing (1.7% in subjects older than 18 years [21] vs. 5.2% in subjects older than 40 years [23]). The prevalence from a nationally representative sample of Chinese adults was 1.7% [10]. The result is considerably lower than those in developed countries, as reported in a large population-based study in Stockholm, Sweden (6.11% in adult) [27], the National Health and Nutrition Examination Survey 2009–2010 (NHANES, 6.5% in adult) [28] and in the Australian Diabetes, Obesity and Lifestyle Study (AusDiab, 11.2% in adults) [29]. The discrepancy might be due to different age groups, different geographic regions or different methods for glomerular filtration rate estimation.

Our study showed that the prevalence of albuminuria in our study was 8.47%, which was similar to the results from the China National Survey of CKD in 2009–2010 (9.5%) [28] and the work of Zhang and colleagues in Beijing (9.2%) [21]. The prevalence of albuminuria in our study were slightly lower than those in Canada [8] (8.47% versus 10.3%). And the prevalence of albuminuria among adults in Poland is 4.5%, which was significantly lower than the result in this study [30]. Potentially, this may be due to the differences in dietary habits and genetic factors between ethnic groups, but further studies are needed.

According to guidelines of KDIGO, CKD severity is classified into five stages on the basis of the GFR levels. Our study showed that prevalence of stages 1, 2, 3, and 4–5 CKD in the elderly population of Qingdao were estimated to be 4.29%, 3.13%, 3.74%, and 0.24%, respectively. It was similar to the results from the China National Survey of CKD in 2007–2010 (5.7%, 3.4%, 1.6%, and 0.13%, respectively) [10]. However, results from NHANES 2009–2010 indicated that prevalence of stages 1, 2, 3, and 4–5 CKD in adults in the United States were 3.2%, 3.1%, 5.8%, and 0.7% [28], respectively. Enyu and colleagues, using data from the Japanese annual health check program for 2000–2004, reported that prevalence of stages 1, 2, 3, and 4–5 CKD were 0.6%, 1.7%, 10.4%, and 0.2%, respectively [31]. It is obvious that the burden of early stages of CKD in China is more serious than those in developed countries. One possible explanation might lie in the differences in age distribution. Many findings suggested that older age was an independent risk factor for CKD, which was further supported by the present study. Although aging has become a prominent social problem in China according to data from the China Population Census in 2010 [4], the percentage of elderly (age ≥ 65) in Qingdao is still less than those in the United States [21] and Japan [32] (10.26% versus 12.3% and 27.9%, respectively). Another possibility might be based on the fact that the prevalence of hypertension and diabetes had rapidly increased in China in the last decades. However, it might take several years or longer for these diseases to cause clinically evident kidney damage at a population level.

In multivariable logistic regression analyses, the results indicated that hyperuricemia was an independent risk factor for the development of CKD, which was consistent with the results of Tae Ryom Oh’s [33] and Srivastava’s [34]. This might be due to the fact that residents’ diets in coastal areas of China are typically high in sodium and potassium due to the high consumption of seafood. However, high sodium intake is significantly associated with rapid decrease in kidney function and increased microalbuminuria [35]. In addition, several studies had showed that beer intake was associated with increased gout and hyperuricemia risks [36,37], thus the high beer consumption in Qingdao may partially explain this phenomenon.

It is interesting to see that OR value of hyperhomocysteinemia was significantly higher than that of other factors in multivariable logistic regression analyses. Simultaneously, the highest prevalence of albuminuria, reduced kidney function, and CKD were observed in subjects with hyperhomocysteinemia, suggesting that hyperhomocysteinemia is a strong independent risk factor for the progression of CKD, which were similar to those in studies of Xu [38], Xie [39], and Li [40] in other parts of China. Potentially, this might be due to the fact that low dietary folate intake and a higher prevalence of MTHFR 677TT mutations in Chinese contribute to the increased frequency of HHcy compared with Western populations [41], but further studies are needed.

Surprisingly, our study showed that the lowest prevalence of albuminuria, reduced renal function, and CKD were present in subjects with diabetes without hypertension, which is contradictive to the results of Zhang [23] and Lee [9]. This may be due to the misjudgments of non-diabetic patients as diabetic patients, because the definition of diabetes was partly based on self-reported history in our study. Additionally, the existence of residual confounding might contribute to the phenomenon.

The prevalence of CKD and reduced renal function were much higher in the females compared with the males. This finding was similar to the results from several other cross-sectional studies [17,42,43]. Several possible explanations of this phenomenon are as follows: (a) Obesity and central obesity may play an indirect role in mediating the pathophysiological development [43]. The prevalence of obesity and central obesity were significantly higher in female (32.3% and 74%, respectively) compared with the male (16.9% and 33.1%, respectively) in this study, which was consistent with the finding of Yu’s [44]; (b) the differences in physiological structure (e.g., glomerular structure and muscle mass) and hormone metabolism between men and women may partly explain this phenomenon [42]; (c) data from the Chinese National Renal Data System (CNRDS) showed that there were more male end-stage renal disease patients with hemodialysis (ESRD-HD) patients than female patients (1.5:1, respectively), and the incidence of ESRD-HD patients obtaining HD was higher in males than in the female population [45]. The result suggested that the access to medical care was substantially limited for women, which might partly explain a higher prevalence of CKD in the females.

Our study showed that exercise was a protective factor for decreased eGFR and CKD. Regular exercise could play an important role in losing weight and slowing down the development of hypertension and diabetes, which are the major risk factors for the progression of CKD. Less exercise might lead to CKD through diabetes and hypertension or via other pathophysiology such as hyperfiltration causing focal segmental glomerulosclerosis [46].

A cohort study showed that each 10 μg/m^3^ increase in the PM_2.5_ (particulate matter with an aerodynamic diameter ≤ 2.5 µm) concentration was associated with a 6% higher risk of developing CKD (HR: 1.06, 95% CI: 1.02–1.10) [47]. A cross-sectional study indicated that traffic-related air pollution were associated with CKD among the elderly population in Taipei city [48]. Bowe et al. [49] also reported that exposure to PM_2.5_ show a significant positive correlation with risk of incident CKD, eGFR decline, and ESRD. In addition, animal studies showed that sub-chronic exposure to PM_2.5_ contributed to early kidney dysfunctions [50]. Above studies indicated that air pollution might be an important risk factor to induce kidney damage. Up to present, it is not clear whether there is a correlation between air pollutants and CKD in Qingdao city and it deserves further investigation.

The study had several limitations. First of all, we only performed a single measurement on all the indicators of blood and urine samples, which might overestimate the prevalence of CKD in the present study. Secondly, this study used a cross-sectional design so it was impossible to infer a causal relationship between CKD and associated risk factors. Finally, we only collected lifestyle information from some of the participants, so we could not determine the role of the factors on CKD.

## 5. Conclusions

In summary, the burden of CKD is relatively high among the elderly population in Qingdao, China. Common chronic non-communicable diseases, including hypertension, diabetes, hyperhomocysteinemia, hyperuricemia, hypertriglyceridemia, and obesity, were associated with greater prevalence of CKD. Considering such prominent effects on CKD progression, specific attentions should be paid to these diseases in any strategy to alleviate the burden of CKD in the elderly in developing countries.

## Figures and Tables

**Table 1:** Demographic and clinical characteristics of the study population.

	Participants with no Indicators of CKD (n = 30,643)	Participants with eGFR < 60 mL/min per 1.73 m^2^ (n = 1377)	Participants with Albuminuria (n = 2929)	Participants with any Indicators of CKD (n = 3945)	Total (n = 34,588)
Age (years)	70.76 ± 6.61	74.70 ± 7.69	72.17 ± 6.91	72.87 ± 7.24	71.00 ± 6.72
**Age group (years)**					
60–69	15,710 (51.3%)	431 (31.3%)	1266 (43.2%)	1578 (40.0%)	17,288 (50.0%)
70–79	11,371 (37.1%)	534 (38.8%)	1167 (39.8%)	1557 (39.5%)	12,928 (37.4%)
≥80	3562 (11.6%)	412 (29.9%)	496 (16.9%)	810 (20.5%)	4372 (12.6%)
**Sex (%)**					
Male	13,385 (43.7%)	490 (35.6%)	1260 (43.0%)	1592 (40.4%)	14,977 (43.3%)
Female	17,258 (56.3%)	887 (64.4%)	1669 (57.0%)	2353 (59.6%)	19,611 (56.7%)
Body mass index (kg/m^2^)	25.80 ± 3.76	26.26 ± 4.18	26.46 ± 3.97	26.38 ± 4.02	25.86 ± 3.80
**Body mass index group (kg/m^2^)**					
<18.5	404 (1.3%)	14 (1.0%)	30 (1.0%)	43 (1.1%)	447 (1.3%)
18.5–23.9	9447 (30.8%)	399 (29.0%)	753 (25.7%)	1049 (26.6%)	10,496 (30.3%)
24.0–27.9	13,137 (42.9%)	568 (41.2%)	1200 (41.0%)	1629 (41.3%)	14,766 (42.7%)
≥28	7655 (25.0%)	396 (28.8%)	946 (32.3%)	1224 (31.0%)	8879 (25.7%)
Central obesity (%)	17,088 (55.8%)	823 (59.8%)	1763 (60.2%)	2378 (60.3%)	19,466 (56.3%)
Diabetes (%)	7540 (24.6%)	378 (27.5%)	745 (25.4%)	1026 (26.0%)	8566 (24.8%)
Hypertension (%)	21,218 (69.2%)	1126 (81.8%)	2406 (82.1%)	3209 (81.3%)	24,427 (70.6%)
Anemia (%)	5439 (17.7%)	527 (38.3%)	630 (21.5%)	989 (25.1%)	6428 (18.6%)
Hyperuricemia (%)	6586 (21.5%)	817 (59.3%)	902 (30.8%)	1499 (38.0%)	8085 (23.4%)
Hyperhomocysteinemia (%)	4124 (13.5%)	712 (51.7%)	721 (24.6%)	1207 (30.6%)	5331 (15.4%)
Exercise (%)	10,086 (32.9%)	345 (25.1%)	902 (30.8%)	1163 (29.5%)	11,249 (32.5%)
Uric acid (μmol/L)	328.22 ± 87.65	415.10 ± 105.96	349.11 ± 100.47	365.35 ± 105.47	332.46 ± 90.63
Homocysteine (μmol/L)	11.85 ± 4.90	17.01 ± 7.47	13.46 ± 6.19	14.27 ± 6.67	12.13 ± 5.19
Total cholesterol (mmol/L)	5.77 ± 1.12	5.90 ± 1.27	5.86 ± 1.24	5.87 ± 1.24	5.78 ± 1.13
Triglyceride (mmol/L)	1.50 ± 0.96	1.84 ± 1.20	1.82 ± 1.35	1.81 ± 1.30	1.54 ± 1.01
LDL cholesterol (mmol/L)	3.39 ± 0.84	3.55 ± 0.94	3.50 ± 0.95	3.51 ± 0.94	3.41 ± 0.85
HDL cholesterol (mmol/L)	1.49 ± 0.30	1.43 ± 0.28	1.46 ± 0.30	1.46 ± 0.3	1.48 ± 0.30
Hemoglobin (g/L)	141.89 ± 13.99	134.10 ± 17.81	141.46 ± 15.94	139.86 ± 16.50	141.66 ± 14.31
Blood urea nitrogen (mmol/L)	5.75 ± 1.48	8.43 ± 3.59	6.62 ± 2.67	6.92 ± 2.75	5.88 ± 1.72
Systolic blood pressure (mmHg)	145.07 ± 22.46	150.76 ± 24.66	153.45 ± 25.08	152.23 ± 24.81	145.89 ± 22.86
Diastolic blood pressure (mmHg)	79.35 ± 12.12	78.85 ± 13.14	81.53 ± 13.29	80.77 ± 13.30	79.51 ± 12.26
Serum creatinine (mg/dl)	0.78 ± 0.18	1.42 ± 0.72	0.91 ± 0.54	1.02 ± 0.54	0.81 ± 0.26
eGFR (mL/min per 1.73m^2^)	103.93 ± 27.43	50.19 ± 10.60	94.07 ± 32.46	83.22 ± 33.78	101.57 ± 28.98

Data were expressed as the mean ± SD or as n (%). LDL—low-density lipoprotein; HDL—high-density lipoprotein; eGFR—estimated glomerular filtration rate; CKD—chronic kidney disease.

**Table 2:** Prevalence of indicators of kidney function in the study population.

eGFR (mL/min per 1.73m^2^)	Kidney Function		Albuminuria		CKD		
	n	Prevalence (95% CI)	n	Prevalence (95% CI)	Stage	n	Prevalence (95% CI)
≥90	20,643	59.68 (59.17–60.20)	1485	7.19 (6.84–7.55)	1	1485	4.29 (4.08–4.51)
60–89	12,568	36.34 (35.83–36.84)	1083	8.62 (8.13–9.11)	2	1083	3.13 (2.95–3.31)
30–59	1294	3.74 (3.54–3.94)	310	23.96 (21.63–26.29)	3	1294	3.74 (3.54–3.94)
15–29	56	0.16 (0.12–0.20)	32	57.14 (43.77–70.52)	4	56	0.16 (0.1–0.20)
<15	27	0.08 (0.05–0.11)	19	70.37 (51.96–88.78)	5	27	0.08 (0.05–0.11)
Total	34,588	100	2929	8.47 (8.17–8.76)	Total	3945	11.41 (11.07–11.74)

Data were expressed as prevalence (95% CI). CI—confidence interval. CKD was defined as eGFR < 60 mL/min per 1·73 m² or albuminuria. eGFR—estimated glomerular filtration rate. CKD—chronic kidney disease.

**Table 3:** Prevalence of indicators of kidney function, by age, sex, and comorbidity.

	Reduced eGFR	Albuminuria	CKD
**Age**			
60–69	2.49% (2.26–2.73)	7.32% (6.93–7.71)	9.13% (8.70–9.56)
70–79	4.13% (3.79–4.47)	9.03% (8.53–9.52)	12.04% (11.48–12.60)
≥80	9.42% (8.56–10.29)	11.34% (10.40–12.29)	18.53% (17.37–19.68)
**Sex**			
Male	3.27% (2.99–3.56)	8.41% (7.97–8.86)	10.63% (10.14–11.12)
Female	4.52% (4.23–4.81)	8.51% (8.12–8.90)	12.00% (11.54–12.45)
**Comorbidity**			
Hypertension	4.61% (4.35–4.87)	9.85% (9.48–10.22)	13.14% (12.71–13.56)
Diabetes	4.41% (3.98–4.85)	8.70% (8.10–9.29)	11.98% (11.29–12.67)
Anemia	8.20% (7.53–8.87)	9.80% (9.07–10.53)	15.39% (14.50–16.27)
Obesity	4.46% (4.03–4.89)	10.65% (10.01–11.30)	13.79% (13.07–14.50)
Hyperuricemia	10.11% (9.45–10.76)	11.16% (10.47–11.84)	18.54% (17.69–19.39)
Hyperhomocysteinemia	13.36% (12.44–14.27)	13.52% (12.61–14.44)	22.64% (21.52–23.76)
Hypercholesteremia	4.60% (4.21–4.99)	9.60% (9.05–10.15)	12.91% (12.29–13.53)
Hypertriglyceridemia	6.80% (6.08–7.52)	13.05% (12.09–14.01)	17.55% (16.47–18.64)
LDL-C ≥ 4.1 mmol/L	5.27% (4.73–5.81)	10.62% (9.87–11.36)	14.32% (13.48–15.17)

Data were expressed as prevalence (95% CI). CI—confidence interval. LDL—low-density lipoprotein; eGFR—estimated glomerular filtration rate; CKD—chronic kidney disease; LDL-C—low-density lipoprotein cholesterol.

**Table 4:** Adjusted odds ratios for indicators of kidney function by risk factors.

Variables	Decreased eGFR		Albuminuria		CKD	
	OR (95% CI)	*p*-Value	OR (95% CI)	*p*-Value	OR (95% CI)	*p*-Value
Age change by 10 years	1.267 (1.105–1.453)	0.001	1.142 (1.048–1.244)	0.002	1.192 (1.104–1.288)	<0.001
Female (vs. male)	1.626 (1.406–1.879)	<0.001	0.845 (0.770–0.927)	<0.001	1.052 (0.968–1.144)	0.233
Central obesity	0.964 (0.842–1.102)	0.590	1.046 (0.956–1.145)	0.327	1.030 (0.951–1.117)	0.468
Obesity	0.925 (0.782–1.093)	0.360	1.251 (1.117–1.400)	<0.001	1.153 (1.043–1.275)	0.006
**Hypertension**						
No indication of disease	Ref	-	Ref	-	Ref	-
Indication of disease and under control	1.818 (1.459–2.265)	<0.001	1.563 (1.327–1.841)	<0.001	1.640 (1.424–1.888)	<0.001
Disease not in control	1.262 (1.084–1.469)	0.003	1.609 (1.452–1.782)	<0.001	1.473 (1.348–1.611)	<0.001
**Diabetes**						
No indication of disease	Ref	-	Ref	-	Ref	-
Indication of disease and under control	1.671 (1.254–2.225)	<0.001	1.551 (1.248–1.928)	<0.001	1.590 (1.317–1.920)	<0.001
Disease not in control	1.558 (1.364–1.778)	<0.001	2.711 (2.496–2.945)	<0.001	2.366 (2.193–2.552)	<0.001
Anemia	2.292 (2.017–2.606)	<0.001	1.235 (1.118–1.364)	<0.001	1.442 (1.324–1.571)	<0.001
Hyperuricemia	3.992 (3.541–4.499)	<0.001	1.237 (1.133–1.350)	<0.001	1.816 (1.685–1.957)	<0.001
Hyperhomocysteinemia	5.645 (4.971–6.409)	<0.001	1.782 (1.614–1.967)	<0.001	2.582 (2.373–2.810)	<0.001
Hypercholesteremia	0.900 (0.765–1.059)	0.206	0.922 (0.827–1.026)	0.137	0.924 (0.840–1.017)	0.108
Hypertriglyceridemia	1.287 (1.108–1.495)	0.001	1.299 (1.172–1.441)	<0.001	1.279 (1.165–1.404)	<0.001
LDL-C ≥ 4.1 mmol/L	1.155 (0.960–1.391)	0.127	1.149 (1.014–1.301)	0.029	1.155 (1.034–1.291)	0.011
Exercise	0.784 (0.687–0.895)	<0.001	0.918 (0.843–0.998)	0.045	0.888 (0.823–0.958)	0.002

Data are multivariable-adjusted odds ratio (95% CI). CI—confidence interval; OR—odds ratio. LDL—low-density lipoprotein; eGFR—estimated glomerular filtration rate; CKD—chronic kidney disease.
